# Socioeconomic cost of AML in Sweden—A population‐based study using multiple nation‐wide registers

**DOI:** 10.1002/jha2.208

**Published:** 2021-05-06

**Authors:** Emma Hernlund, Josefine Redig, Björn Paulsson, Åsa Rangert Derolf, Martin Höglund, Simona Vertuani, Gunnar Juliusson

**Affiliations:** ^1^ ICON plc Stockholm Sweden; ^2^ Novartis Oncology Nordics Stockholm Sweden; ^3^ Swedish Acute Myeloid Leukemia Registry Group; ^4^ Division of Hematology Department of Medicine Karolinska University Hospital, Solna, Karolinska Institutet Stockholm Sweden; ^5^ Department of Medical Sciences Uppsala University Uppsala Sweden; ^6^ Departments of Hematology and Stem Cell Transplantation Skåne University Hospital Lund Sweden

**Keywords:** acute leukemia, cost, national registers, treatment

## Abstract

Acute myeloid leukemia (AML) is associated with a high economic and clinical burden. Recently novel therapies have been added to standard treatment regimens. Here, we evaluated the economic impact of AML up until the introduction of these novel therapies. Individual data on 2954 adult patients diagnosed from 2007 to 2015 from five Swedish national population‐based registers were used, enabling analyses from diagnosis to either death or 5‐year follow‐up for survival, inpatient and outpatient costs, costs of prescribed drugs, sick leave, and early retirement. Costs per patient were stratified by age group, treatment options, and *FLT3*‐ITD status. The expected 5‐year costs per patient differed substantially between age groups. Patients aged 18–59 years had an expected mean cost per patient of €170,748, while age groups 60–69 years, 70–79 years, and >80 years incurred an expected mean cost of €92,252, €48,344, and €24,118, respectively, over 5 years. Patients <60 years undergoing stem cell transplantation had the highest costs (€228,525 over 5 years). About 60% of costs for these patients were from hospitalizations and 20% from sick leave and early retirement; cost per day was highest from the first admission to complete remission. This study provides a baseline for socioeconomic evaluations of novel therapies in AML in Sweden.

## 1 INTRODUCTION

Acute myeloid leukemia (AML) is a disease with poor prognosis, causing 85,000 deaths globally in 2016. This was estimated to cause 2.6 million life‐years loss, leading to high costs for individuals, families, and society [1]. AML is diagnosed at all ages, from newborn to elderly; however, the incidence rises sharply in older age [2, 3, 4].

Intensive chemotherapy has been based on cytarabine and daunorubicin since the 1970s [5, 6, 7]. This has been the recommended treatment option in Sweden for most patients aged ≤80 years, leading to complete remission (CR) rates of 50–90%[5], depending on age [3, 8]. However, relapse rates remain high. Allogeneic stem cell transplantation (alloSCT) is estimated to halve the risk of relapse [9] and is therefore indicated for patients with high or intermediate genetic risk [5], such as those with internal tandem duplication (ITD) of the FMS‐like tyrosine kinase 3 (*FLT3)* gene (*FLT3*‐ITD) [10]. The transplant rate is increasing [11], especially in patients aged >60 years [8], due to the development of better‐tolerated conditioning, improved tissue typing, and expanded registers for voluntary stem cell donors [12, 13].

For many years, the cost of anti‐leukemia drugs has been low due to the lack of novel therapeutics. On the other hand, the severe toxicity from intensive chemotherapy leads to long and repeated hospitalizations and the need for extensive supportive care including blood transfusions and anti‐infective drugs. There are also high expenses associated with alloSCT, including searches for and cell harvests from suitable donors.

During the last decade, older patients and/or those with comorbidities have been treated with hypomethylating agents (HMAs) [14, 15, 16]. Although HMAs are more expensive, and complete remission(CR) rates and overall survival are low, their relatively low toxicity enables treatment in an outpatient setting. A significant proportion of elderly patients receive palliation only, with lower drug costs and shortened residual life span [3, 8].

Recently, a number of new targeted and nontargeted oral therapies, such as *FLT3* inhibitors, with low toxicity have been developed [17, 18] showing efficacy as monotherapy or in combination with chemotherapies or HMAs. These drugs will likely be used in various treatment combinations, adding to costs of drug therapy, but also leading to improved outcomes for patients.

Studies addressing the cost and burden of AML are sparse; few of these report costs outside the US setting [19, 20, 21, 22, 23, 24, 25, 26, 27], and none are population based. This study provides a baseline for economic evaluations of novel therapies in AML in Sweden.

## 2 METHODS

### 2.1 Study design

The study population in this noninterventional retrospective register study consists of adult patients diagnosed with AML in Sweden between January 1, 2007, and June 30, 2015. Eligible patients were identified in the Swedish Cancer Register (SCR) with International Classification of Diseases for Oncology, 3rd Edition (ICD‐O/3) morphology codes (SNOMED3) for AML (98963, 98713, 98973, 98723, 98733, 98743, 98673, 98913, 98403, 99103, 98953, 98703, 99313, 99303, 98613, and 99203). Patients diagnosed at autopsy were excluded.

The index date was defined as the diagnosis date in the SCR, the date of the sampling of the first diagnostic test defining AML. Analyses were performed in the postindex period, which varied in length from a minimum of 6 months to a maximum of 9 years. Subjects were right‐censored at end of follow‐up defined as 5 years after the date of diagnosis or end of data availability, whichever occurred first.

In the analyses of costs per treatment phase, a start event (e.g., first CR) and an end event (e.g., relapse, SCT, death, or end‐of‐data, whichever occurred first) were defined, and total costs and mean length of the respective treatment phase were estimated.

Information on *FLT3*‐ITD mutation was recorded in the Swedish National AML Registry (SwAMLR). It should be noted that other *FLT3* mutations, such as point mutations, were not recorded.

### 2.2 Data sources

Data were retrieved from five national registers: the SCR, the National Patient Register (NPR), the Prescribed Drugs Register (PDR) (all held by the National Board and Health and Welfare), Micro‐Data for the Analysis of Social Insurance (MiDAS) (held by the Swedish Social Insurance Agency), and the SwAMLR (held by the Swedish AML Group and the Regional Cancer Center South). After identification in the SCR, data on resource use were extracted for all subjects from the NPR (inpatient and outpatient), PDR (prescribed drugs), and MiDAS (sick leave and early retirement). Data on disease characteristics, treatment, and disease progression were extracted from the SwAMLR. Anonymized data were linked using personal identification numbers unique to every Swedish resident. The SwAMLR collects information on all adult Swedish residents with an AML‐diagnosis since 1997; the coverage is >98% compared to the SCR. In this study, we analyzed data on patients diagnosed from 2007 to mid‐2015 to include patients’ primary therapy with sufficient follow‐up [8]. Patients were treated according to Swedish National Guidelines [8, 28].

### 2.3 Analysis

Survival was analyzed using standard Kaplan–Meier survival estimations. Hospital‐care resource use was costed using diagnosis‐related group (DRG) remunerations, including costs of inpatient drugs. Costs for prescribed drugs were derived directly from the PDR. The cost of sick leave and early retirement were estimated by applying the Swedish mean daily salary on the estimated number of days of sick leave and early retirement. Only visits and prescriptions categorized as AML related were included. Hospital resource use was categorized as AML related based on AML‐specific diagnoses and/or type of hospital and/or clinic. Prescribed medications related to the treatment of AML were defined by the specialist training and/or care setting of the prescriber. Sick leave and early retirement were classified as AML related if they were associated with an AML diagnosis code or toxicity due to transplantation. AML diagnoses, specialist training, and hospital clinics included capturing AML‐related resource‐use were ICD‐10 codes C90, C92, C93, C94, C95, C96, D46, and T86, hematologists or oncologists, and hematology clinics or oncology clinics.

For the analyses of expected costs, time was divided into 30‐day intervals starting from the index date. Mean costs per patient with follow‐up time were estimated per month for all cost categories and expected 5‐year costs were estimated by multiplying mean costs with survival estimates for each month, using the method described by Lin et al. to account for right censoring [29]. Finally, resource use and costs were summed to estimate the total accumulated expected cost over 5 years. For the cost of HMAs, it was assumed that patients were on treatment for 75% of the time (135 mg per day for 7 days per month).

For the analyses of costs per treatment phase, total mean costs were divided by total mean days spent in the treatment phase to estimate the mean cost per day in each treatment phase.

All costs were inflated to 2018 Swedish Krona (SEK) using the healthcare component of the price index and then converted to Euros (1 Euro = 10.26 SEK). All analyses were conducted in Stata version 15 (StataCorp LLC, TX, USA).

## 3 RESULTS

A total of 2954 adult patients aged ≥18 years were identified with an AML diagnosis in the SCR. A detailed description of the patient population, including subgroups, is presented in Table 1.

**TABLE 1:** Demographic characteristics of acute myeloid leukemia (AML) population and subgroups shown in Figures 1, 2, 4

				Treatment			
Group	*N*	Mean age (median age)	Patients undergoing aSCT (%)	Intensive treatment (%)	Hypomethylating treatment (%)	Palliative treatment (%)	Unknown treatment (%)
All	2954	68.2 (71)	17.5	60.0	3.9	33.6	2.5
Age							
18–59 years	680	45.5 (49)	53.1	93.2	1.2	2.9	2.6
60–69 years	670	64.9 (65)	22.2	84.2	2.2	11.3	2.2
70–79 years	869	74.6 (75)	0.8	58.2	6.2	33.1	2.4
80+ years	735	84.7 (84)	0.0	9.3	5.0	83.0	2.7
Treatment							
Intensive	1772	61.1 (64)	28.3	100	–	–	–
Hypomethylating	114	74.8 (76.5)	–	–	100	–	–
Palliative	994	80.0 (81)	–	–	–	100	–
Transplantation							
aSCT	261	43.1 (46)	100	100	–	–	–
No aSCT	201	47.0 (51)	–	100	–	–	–
Survival							
<1 year	791	74.1 (77)	3.2	41.7	0.8	54.7	2.8
1–5 years	301	65.2 (68)	24.3	80.7	2.7	15.3	1.3
>5 years	247	52.7 (56)	51.0	97.2	0.4	1.2	1.2
FLT3‐ITD mutation							
Negative	899	60.5 (63)	28.0	88.3	0.2	8.9	2.6
Positive	298	58.6 (61)	39.6	90.3	0.3	8.4	1.0

Overall, patients had a mean (median) age of 68.2 (71) years at diagnosis; 48% were female. A majority of patients were treated with high‐dose chemotherapy (60%), whereas approximately 34% received palliative treatment up‐front. Approximately one in six patients underwent SCT as part of their treatment.

### 3.1 Expected costs by age groups

**FIGURE 1:**
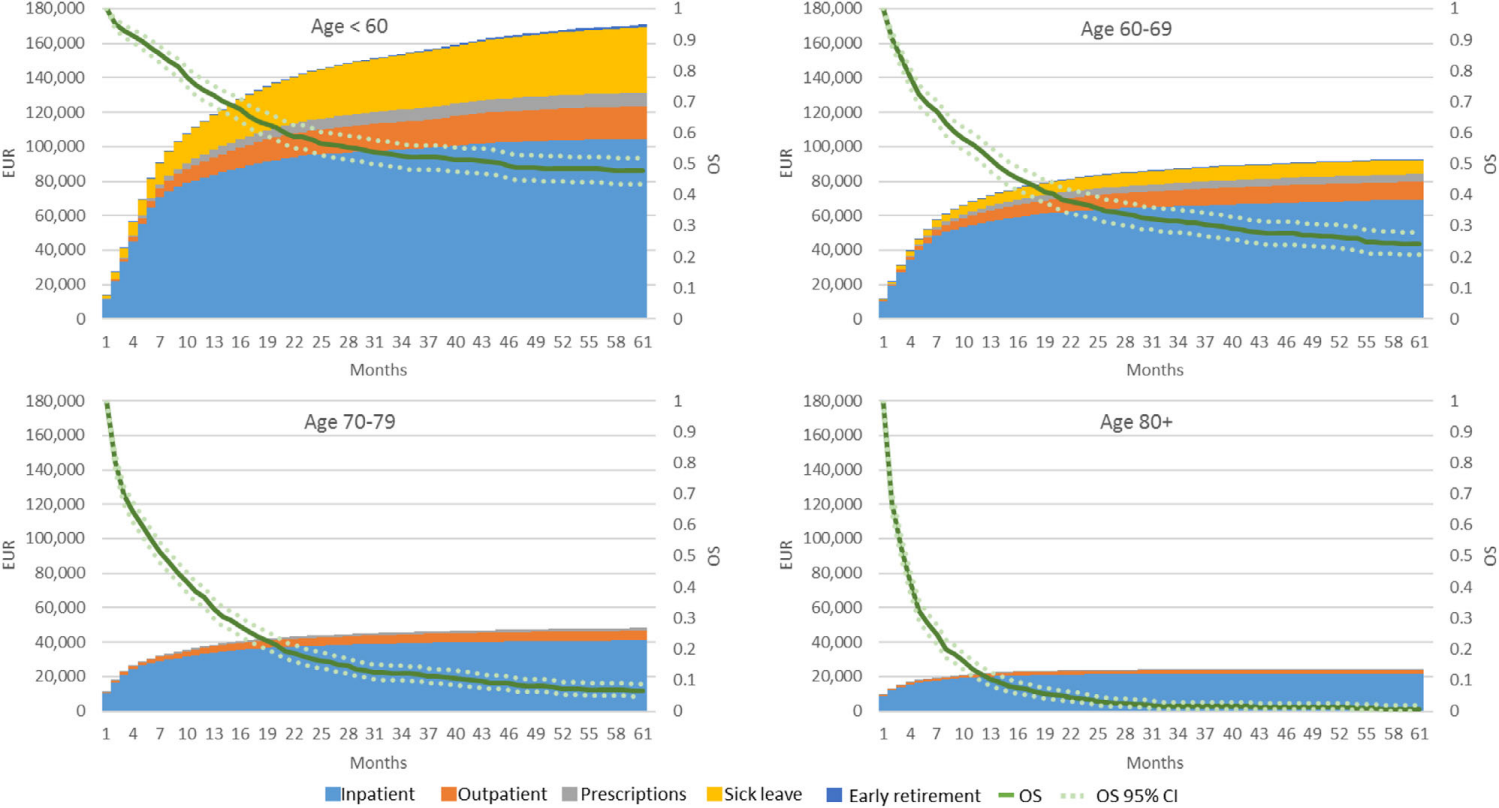
Accumulated expected costs per time period for age groups

Overall survival and accumulated expected costs per time period, by age group, are presented in Figure 1. Over 5 years, patients diagnosed with AML below the age of 60 had an expected cost of €170,748 per patient and an estimated overall survival of 48%. The largest proportion of costs was attributable to inpatient resource use, where nearly 60% of the costs was incurred during the first 6 months and 80% in the first 12 months. Regarding costs due to work absence, approximately 50% of the costs due to sick leave was acquired in the first 12 months, whereas only 13% of the costs due to early retirement was acquired during the same time period.

In older age groups, overall survival and the total accumulated 5‐year cost per patient decreased, whereas the proportion of costs acquired in the first 12 months increased. The expected 5‐year cost per patient for patients aged 60–69 and 70–79 years at diagnosis was €92,252 and €48,344, respectively, and overall survival at 5‐years was 5.7 and 6.4%, respectively. For the eldest age group (aged ≥80 years), the expected 5‐year cost per patient was €24,118. For these patients, 90% of the total 5‐year expected cost per patient was due to healthcare resource use in the first 12 months after diagnosis, and overall survival was less than 1% at 5 years.

### 3.2 Expected costs by treatment

**FIGURE 2:**
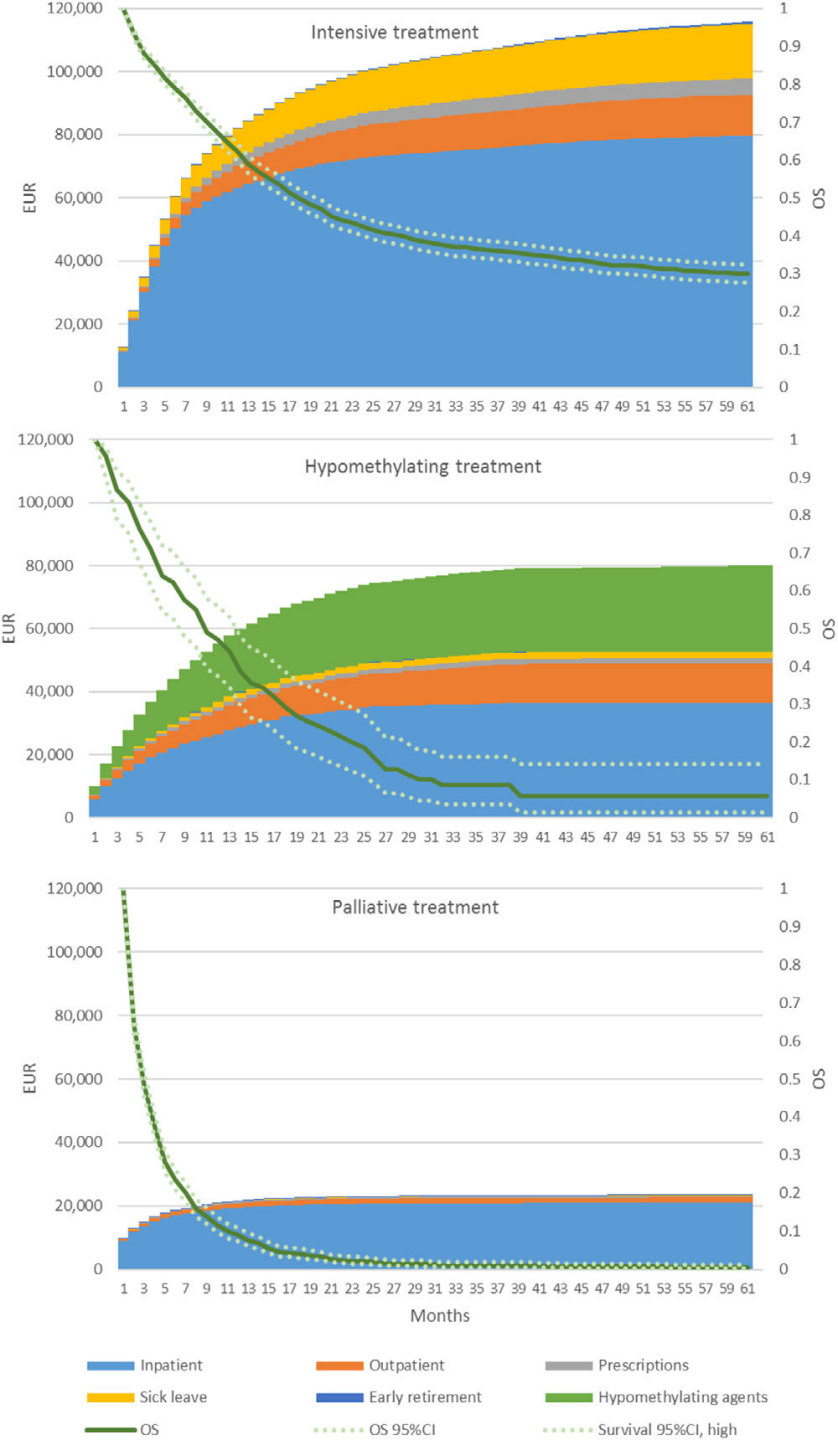
Accumulated expected costs per time period by treatment

In Figure 2, accumulated expected costs and overall survival restricted to the 5‐year follow‐up are stratified by treatment as reported in the SwAMLR. Since HMAs are mostly administered in an outpatient setting, these are not properly reflected in the costing approach using DRG remunerations. The costs of these drugs were therefore estimated separately.

The total expected costs over 5 years varied by treatment; the expected 5‐year costs per patient with intensive treatment amounted to €115,830, while the corresponding cost per patient treated with HMAs was €80,010 (€52,648 excluding the assumed cost of HMAs) and €23,291 for patients undergoing palliative treatment. Similarly, overall survival for the treatment groups varied with median survival estimates of 16.8, 9.8, and 1.9 months for patients with intensive treatments, HMAs, and palliative treatments, respectively. The cost of outpatient visits was similar for patients receiving intensive treatments and HMAs, as expected 5‐year costs per patient amounted to €12,965 and €12,395, respectively. Costs of sick leave were negligible except for patients with intensive treatments, as patients in this group were comparably younger (Table 1).

### 3.3 Expected costs for alloSCT and non‐SCT patients up to 60 years of age

**FIGURE 3:**
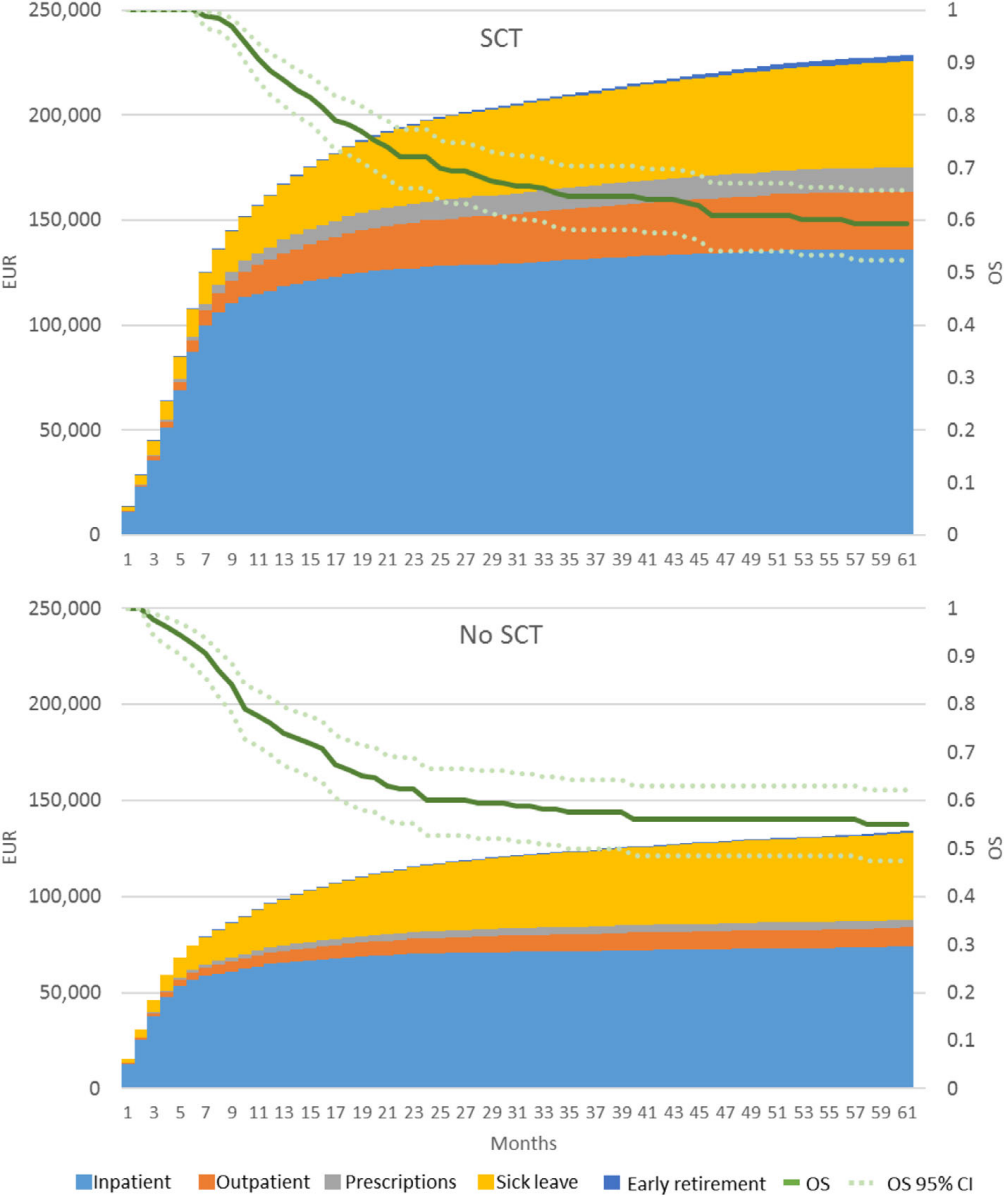
Accumulated expected costs per time period for patients <60 years achieving complete remission stratified by stem cell transplantation (SCT) within 1 year of diagnosis or no SCT

The subgroup of patients <60 years achieving first CR after high‐dose chemotherapy and undergoing alloSCT within the first year, and before the first relapse, was stratified by SCT status for the analysis presented in Figure 3. Patients undergoing SCT within 1 year from diagnosis were compared to patients not undergoing SCT. Accumulated expected 5‐year costs per patient were €228,525 and €133,851 for patients undergoing SCT and not undergoing SCT, respectively. In terms of absolute costs, inpatient costs for patients undergoing SCT were almost twofold compared to patients not undergoing SCT. The costs in the first 6 months were comparable to inpatient costs; thereafter inpatients costs for the patients undergoing SCT escalated. This coincided with the point in time from diagnosis when, on average, SCT was performed. The mean (median) number of days from diagnosis to SCT in the first CR was 148 (142), ranging from 60 to 352 days. For costs attributable to outpatient visits, prescribed medication and early retirement costs were approximately threefold for the group of patients undergoing SCT compared to those not undergoing SCT. After 24 months, the cumulative cost for SCT patients increased more than for non‐SCT patients.

### 3.4 Expected costs by *FLT3* mutational status

**FIGURE 4:**
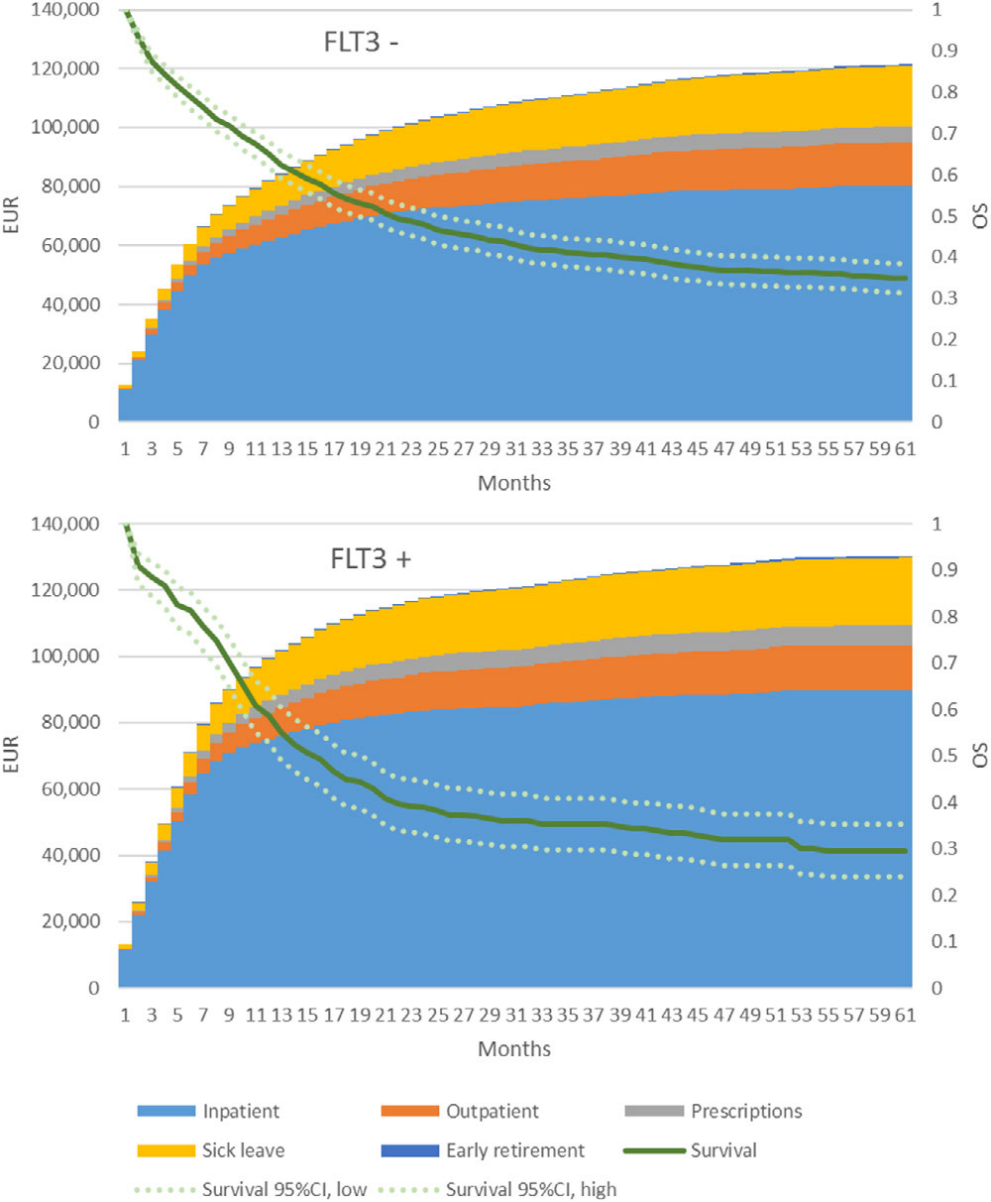
Accumulated expected costs per time period for patients tested positive and negative for *FLT3*‐ITD mutation

*FLT3*‐ITD mutational status was analyzed in 1197 patients. Out of these, 899 patients (75%) were negative for the mutation and 298 patients (25%) were positive for the mutation. The age distribution and treatments were similar between patients with *FLT3* positive and negative AML, with the exception of more SCTs being performed in the *FLT3*‐positive patient group (see Table 1). Expected accumulated costs were slightly higher in the *FLT3*‐ITD positive group (Figure 4). The greatest difference in total costs was observed in the first year after diagnosis, corresponding to the period when the majority of SCTs were performed. Overall survival was lower in *FLT3*‐ITD patients versus *FLT3*‐ITC negative patients (5‐year OS 29.5% [95% CI 23.9 – 35.2%] vs. 34.9% [95% CI 31.3–38.4]). This means that fewer patients in the *FLT3*‐ITD positive group contributed to costs over time.

### 3.5 Expected costs by survival time

**FIGURE 5:**
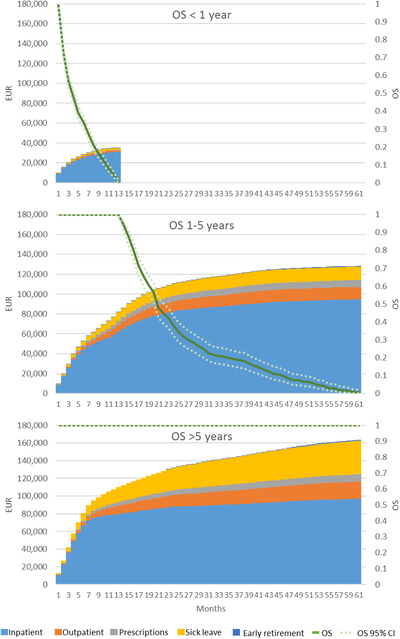
Accumulated expected costs per time period for patients diagnosed from 2007 to 2010 stratified by overall survival

In Figure 5, results for patients diagnosed between January 1, 2007 and December 31, 2010 are stratified based on overall survival. Patients who did not survive more than 1 year had accumulated expected costs estimated at €35,034 per patient. Accumulated expected 5‐year costs for patients surviving between 1 and 5 years were estimated at €128,308 per patient. The subgroup of patients who had an observed survival after 5 years had an accumulated expected cost of €163,853 per patient, with a high proportion of patients undergoing SCT (approximately 50%). Even though all patients surviving between 1 and 5 years, as well as >5 years, were alive in the first year, total costs differed between the two groups. After 6 months, total costs amounted to €57,826 in patients surviving 1–5 years and €89,636 in patients surviving >5 years; at 1 year the total costs amount to €81,592 and €110,027, respectively.

### 3.6 Costs by treatment phase

In addition to the analyses of expected accumulated cost from diagnosis until 5 years thereafter, analyses of costs in different treatment phases were performed.

The analysis from the first admission to CR included 1243 patients. The mean number of days from first day of the first admission to CR was 45 and mean total costs during this time amounted to €23,086. For patients with intensive treatment not achieving CR, mean total costs amounted to €26,294 over a mean of 63 days. For these patients, the treatment phase was assumed to end 90 days after the first day of first AML admission, at death, or SCT, if they occurred before 90 days.

For patients achieving CR, costs were estimated until relapse, death, SCT, or end of the follow‐up to a maximum of 5 years. Out of the 1243 included in the analysis from the first admission until CR, six patients were excluded from subsequent analyses due to lack of follow‐up after achieving CR. This treatment phase had a mean duration of 439 days and included long‐term survivors. Total mean costs over this period were estimated to €43,040 per patient.

The analyses of costs from relapse to death or end‐of‐data were separated into patients receiving palliative treatments after relapse and patients treated with re‐induction high‐dose chemotherapies. The mean number of days for patients treated with palliative care after relapse was 152 days, and 398 days for patients with re‐induction treatments. Total mean costs were estimated at €32,925 and €59,942, respectively.

Of the 350 patients receiving re‐induction treatment after relapse, 178 patients were recorded as having a second CR. These patients were followed from second CR until death, end‐of‐data, or a maximum of 5 years, resulting in a mean of 585 days. Mean total costs were estimated to €99,609 during this time.

The highest total mean cost was found in patients undergoing SCT. Patients were followed from the day of transplantation until death, end‐of‐data, or a maximum of 5 years for a mean of 844 days. The total mean cost incurred was estimated to €137,209 per patient.

**FIGURE 6:**
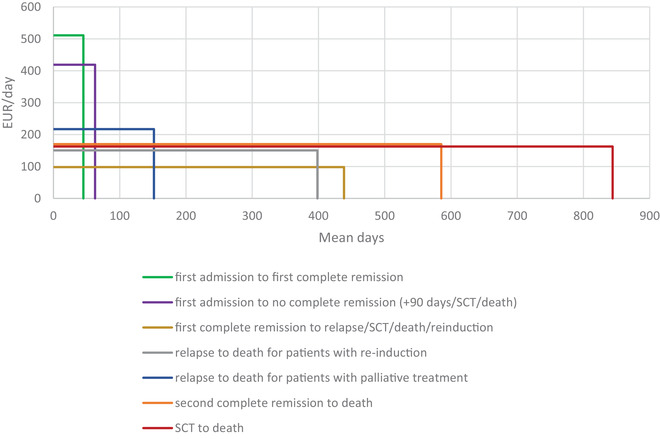
Cost per day in treatment phase and treatment phase duration

The mean cost per day and mean number of days for the respective treatment phases are depicted in Figure 6. The area under the curve represents the total cost during the treatment phase. As expected, costs were higher during induction treatment at the beginning of the treatment course. Total costs were found to be highest when resource use and associated costs were incurred over a longer time, as observed in patients undergoing SCT.

## 4 DISCUSSION

Here, we show that for 2954 patients, representing all patients diagnosed with AML in Sweden from 2007 to 2015, the mean expected cost per patient treated with high‐dose chemotherapy, HMA, and palliative care only was €115,830, €80,010, and €23,291, respectively. The major finding of this study is that costs are driven by hospitalizations (including for alloSCT), mainly due to the administration of high‐dose chemotherapy treatments, as well as treating complications due to drug toxicity, including febrile neutropenia.

One limitation of the study is related to study design—the studied population did not have the same follow‐up time, which limits comparisons between patients. A second limitation relates to the data sources utilized in this study. Hospital resource use was estimated from the NPR, which does not record visits to healthcare professionals other than medical doctors, thus underestimating total costs from health care interactions. As only resource use associated with diagnosed patients is included, costs associated with donor stem‐cell donations and assistance from relatives and caregivers are not included in this analysis. In addition, the costing approach applies remuneration values for DRG codes associated with each stay or visit; these costs are estimated from nonoutlier costs. In general, the inclusion of outlier costs is estimated to increase DRG remunerations by approximately 20% [30]. Thus, the costs of hospital resource use are most likely underestimated.

The majority of studies reporting costs of AML treatment have estimated costs over a limited time period (e.g., 6 months or 1 year [19, 25, 31]) or for a small patient population (e.g., single‐center study‐population, only insured patients [19, 22, 23, 27]). Existing literature mainly describes costs of AML in the US setting [19–21, 23, 25, 26]. The cost of healthcare is generally higher in the United States than in other comparable countries, and treatment costs in the United States differ heavily depending on insurance status [21, 23, 25, 26, 31].

To our knowledge, this study is unique in its nationwide population‐based approach, covering different patient subsets as well as treatment phases. It was clear that the economic costs of AML are substantial, even with few new drugs introduced during the study period. Healthcare systems moving treatments to the outpatient setting, including home care, are likely to reduce costs [32]. If newer therapies can induce and maintain remission with less toxicity and comparable efficacy to existing treatments, at least in subsets of AML patients, substantially increased drug costs could be offset and a major improvement of patient quality of life could be possible. The ongoing development of novel AML therapies thus has the potential to be beneficial not only for AML patients, their families, and caregivers but also for health care providers and the wider society. This study can enable future comparisons of novel AML therapeutics to the therapeutics included in this study, allowing for the examination of contrasts between novel and older therapeutics in costs by age, type of treatment, and treatment phase.

## AUTHOR CONTRIBUTIONS

Emma Hurnlund designed the study together with Simona Vertuani, Josefine Redig, Björn Paulsson, and Gunnar Juliusson. Åsa Rangert Derolf and Martin Höglund had critical insights in the interpretation and validation of the results of the study and helped in drafting the manuscript. All authors contributed to the analysis of the data and interpretation of the results.

## CONFLICT OF INTEREST

Gunnar Juliusson has participated in advisory boards for AbbVie, Astellas, Astex, Celgene, Daiichi Sankyo, Jazz Pharma, Merck, Novartis, and Sunesis. Josefine Redig and Emma Hurnlund, employees of ICON, were paid consultants to Novartis for development of this manuscript. Simona Vertuani and Björn Paulsson are Novartis employees.

## Data Availability

Data cannot be shared due to restrictions in ethical permission and data privacy regulations from the data holders.
